# Modeling the Effect of Optical Signal Multipath

**DOI:** 10.3390/s17092038

**Published:** 2017-09-06

**Authors:** Álvaro De-La-Llana-Calvo, José Luis Lázaro-Galilea, Alfredo Gardel-Vicente, David Rodríguez-Navarro, Ignacio Bravo-Muñoz, Georgios Tsirigotis, Juan Iglesias-Miguel

**Affiliations:** 1 Department of Electronics, University of Alcalá, Alcalá de Henares, 28801 Madrid, Spain; alvaro.llana@uah.es (A.D.-L.-L.-C.); alfredo.gardel@uah.es (A.G.-V.); david.rodriguezn@edu.uah.es (D.R.-N.); ignacio.bravo@uah.es (I.B.-M.); juan.iglesiasm@edu.uah.es (J.I.-M.); 2 Computer and Informatics Engineering Department, Eastern Macedonia and Thrace Institute of Technology, 65404 Kavala, Greece; tsirigo@teiemt.gr

**Keywords:** infrared, multipath, reflection modeling, impulse response, indoor optical wireless, indoor positioning system

## Abstract

Here, we propose a model to determine the effect of multipath in indoor environments when the shape and characteristics of the environment are known. The main paper goal is to model the multipath signal formation to solve, as much as possible, the negative effects in light communications, as well as the indoor positioning errors due to this phenomenon when using optical signals. The methodology followed was: analyze the multipath phenomenon, establish a theoretical approach and propose different models to characterize the behavior of the channel, emitter and receiver. The channel impulse response and received signal strength are obtained from different proposed algorithms. We also propose steps for implementing a numerical procedure to calculate the effects of these multipaths using information that characterizes the environment, transmitter and receiver and their corresponding positions. In addition, the results of an empirical test in a controlled environment are compared with those obtained using the model, in order to validate the latter. The results may largely vary with respect to the cell size used to discretize the environment. We have concluded that a cell size whose side is 20-times smaller than the minimum distance between emitter and receiver (i.e., 10 cm × 10 cm for a 2-m distance) provides almost identical results between the empirical tests and the proposed model, with an affordable computational load.

## 1. Introduction

Wireless communications in indoor environments have witnessed substantial consolidation, especially those based on optical communications using infrared or visible signals (VLC). They provide secure communications, because the signal is confined indoors, and work with high bit rates. As lighting is now based on LEDs and this type of device can work at high frequencies, it is now possible to illuminate and send information simultaneously. Consequently, lighting systems can also be used for communications and to implement indoor local positioning systems (ILPS).

In optical communications in closed environments, higher bit rates are being achieved thanks to advances in the technologies employed to manufacture new devices (transmitters and receivers), which present higher bandwidths and thus permit the use of different types of modulation. Various positioning methods based on emitting/receiving optical signals use trilateration from distance measurements obtained from time of flight (ToF), phase of arrival (PoA) [1,2] and received signal strength (RSS) [3]. Position can also be estimated via triangulation by measuring the angle of arrival (AoA) at which the signal reaches the receiver.

One of the main barriers to implementing communications systems and ILPS, whether using ad hoc systems or leveraging the available infrastructure, is the effect of multipath (MP) signal propagation. In communications, signals degrade, and intersymbol interference (ISI) increases, substantially reducing the bit rate; in ILPS, independently of the technology used, multipath may give rise to distance measurement errors of metric magnitudes, rendering it impossible to use an ILPS system without deploying multipath mitigation techniques. Due to this drawback, it is of fundamental importance to develop a methodology and procedure with which to estimate the effect and error generated by multipath in a working environment with a given geometry. This would eliminate the need to implement systems to verify the error and would permit comparisons between different alternatives in order to implement the best and even mitigate the errors in a next step.

Here, we present a proposal for a procedure and algorithms that make it possible to introduce the characteristics of the environment (e.g., models of the reflection from its different parts, geometric shape, size, conditions and constraints), the transmitter and the receiver and use this information to obtain the impulse response due to multipath generated by different reflections. This tool thus facilitates the study and analysis of multipath behavior.

The proposed composite signal (MP)model and procedure are based on the material reflection model given in [4], which was also developed for this purpose.

## 2. Background

Several studies have developed algorithms and methods to estimate the channel impulse response due to multipath. Of these, one in particular merits special mention. Although developed some time ago, the method described in [5] has been cited in many articles and is still used today. It consists of a recursive method that estimates the impulse response by calculating the signal strength received from the source in each part of a discretized environment. These parts are then considered new transmitters that emit from a central point on each part, discretized using a Lambertian pattern.

The present proposal follows the same procedure up until a number *K* of reflections. The main problem with this method is that it considers all reflections as Lambertian transmitters, regardless of the materials in the environment or angles of arrival, etc. However, as shown in [4], this is far from the case in reality. Although this study is not recent, it presents an intuitive approach to discretizing the environment in order to work with current numerical methods and tools. However, in practice, it would only be applicable in environments with materials presenting very diffuse reflection (almost perfect diffusion), which is rarely the case. The simplicity of the reflection model employed renders it relatively easy to obtain a model of the signal generated by multipath because the angle of arrival in the environment of the wavefront of the transmitter signal is not considered at any time; however, it does not model reality correctly. When the model of signal reflection from the materials considers various components and the behavior of each depends on the angle of arrival, the final model is very complicated. This difficulty increases exponentially with the number of rebounds (reflections) considered.

In [6], a method is described for estimating the impulse response using multiple input multiple output (MIMO) in a matrix, dividing the parameters of the transmitter, environment and receiver into different matrices. This procedure facilitates calculations, which can be performed by sweeping in a single matrix, for example, to analyze the effects of multipath with different transmitter positions in the environment.

The above recursive methods impose a large computational load. Thus, methods based on Monte Carlo simulations (MMC) have also been proposed to obtain the impulse response due to multipath, which require a much lower load [7,8,9]. These use probability densities and are less accurate than recursive methods.

Combining the advantages of the above two methods, [10] proposed a method that calculates the first reflection deterministically, as in [5], but subsequent reflections are calculated using MMC methods. This approach obtains good accuracy for the first reflection and enables rapid calculation of a large number of rebounds.

There are several studies that analyze the effects of MPs, in communications and LPS systems, based on the impulse response [11,12,13,14]. The work in [15] analyzes the impact of high order light reflections on indoor optical wireless communication.

Most studies on calculating the impulse response due to the multipath of the optical signal consider surface reflection in the environment as Lambertian or the Phong reflection model [16] in more complex methods; however, as can be seen in [4], this behavior does not represent the reality of most materials.

Here, we present a method to estimate the channel impulse response based on the combined advantages of the methods described above, but considering that the reflection from materials is not Lambertian, but rather follows a reflection model such as that proposed in [4]. Using this model greatly complicates the proposal for a tool to characterize multipath behavior, but it is the only means to deduce the real behavior and make decisions with the results obtained.

It is important to note that our model considers the signal strength of each path and its corresponding phase, which will depend on the path taken. Lastly, the type of sensor used and the intended application will affect the necessary information to take into account.

To validate the proposal and provide application examples, we performed an empirical test in a controlled environment and then a simulation using our model under the same conditions as in the empirical test. The aim of this step was to validate our model, showing that the result obtained for the multipath effects is similar to that obtained empirically.

## 3. Theoretical Approach

### 3.1. Initial Considerations

The channel impulse response is formed by the infinite contributions of the multipath caused by light reflections in the environment. Since the indirect paths are infinite, we chose to discretize the environment. To do this, we divided all of the walls, the floor and the ceiling of the environment into a grid of cells. The signal strength reaching each of the cells is calculated from the energy emitted by the transmitter. To model reflection, each of the cells is considered a one-off transmitter that emits to the rest of the cells from a point at its center, according to a given reflection model. These in turn emit to the rest and so on successively. Each emitted (reflected) signal is associated with the signal strength and phase parameters, which are used to calculate the impulse response.

To assume this model, which receives energy in an area, but emits from a point, the irradiance received throughout the area must be approximately constant (discretisation of the environment yields results very similar to the continuous case). In order to consider that the irradiance reaching each point of the cell is constant, the cell size must be much smaller than its distance to the transmitter; in optoelectronics, it is usually assumed that irradiance is constant if the solid angle covered is less than 0.01 sr. To calculate the solid angle that covers each of the grid cells and verify that the estimates are feasible without altering the final result, the following expression is used:

(1) $$d\Omega =\frac{dS\cos\left(\theta \right)}{R^{2}}$$

where *R* represents the distance from the transmitting source to the cell center and θ is the angle formed by the vector that joins each dS with the surface vector of the source.

Assuming the solid angle covers a spherical cap of area *S* (or a circle of area *S* if r>>S), the equation is expressed as:

(2) $$\Omega =\frac{S}{r^{2}}$$

Since the environment is discretized, time must also be discretized to calculate the impulse response. In [5], it is established that a good choice of time interval is $\Delta t=\sqrt{S}/c$, which would be the time it takes light to travel between two neighboring cells.

To implement our proposal, the source, the receiver and each grid cell will be termed an element, and each of these elements will be characterized by the parameters “position” and “surface vector”. In addition, the source and receiver elements will be associated with the parameters of transmission and reception, respectively, and the elements forming the grid will be associated with the parameters of the reflection model for the material forming each element in the environment.

As regards the nomenclature used in this paper, the array $r_{x}$ represents the coordinates of the element *x*, and the vector ${\vec{v}}_{x}$ is the surface vector of the element *x*.

### 3.2. Transmitter and Receiver Model and Line of Sight Path

**Figure 1:**
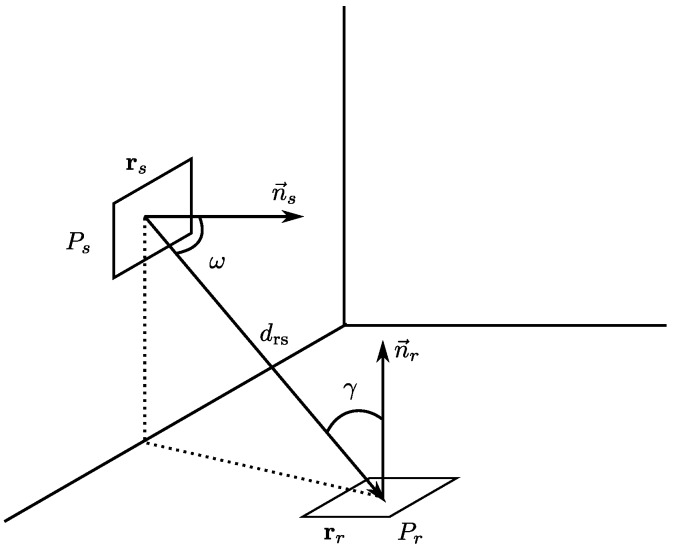
Diagram of the components of the received signal strength at a point *x* ($P_{x}$).

Assuming an ideal channel, the signal strength emitted is stable, and the signal strength received by a detector via the LOS path $P_{\mathrm{rs}}$, in relation to the energy emitted by the transmitter (Figure 1), can be expressed according to Equation (3):

(3) $$P_{\mathrm{rs}}=I\left(\omega \right)\frac{1}{d_{\mathrm{rs}}^{2}}F\left(\gamma \right)R\left(\gamma \right)A_{r}=E\left(\omega \right)F\left(\gamma \right)R\left(\gamma \right)A_{r}$$

where $I\left(\omega \right)$ represents the energy output function of the transmitter (emission pattern), $d_{\mathrm{rs}}$ is the distance between the source and the receiver, $F\left(\gamma \right)$ is the transmission function of a possible filter placed on the receiver, $R\left(\gamma \right)$ is the receiver response (which includes the gain of possible concentrators and its response) and $A_{r}$ is the active area of the receiver. $E\left(\omega \right)$ represents the energy per surface unit that the transmitter generates at the point where the detector is located.

If the emission pattern is represented by a generic exponential emission equation, $I\left(\omega \right)$ can be represented as:

(4) $$I\left(\omega \right)=\frac{n_{s}+1}{2\pi }P_{s}\cos^{n_{s}}\left(\omega \right)$$

where $n_{s}$ is the index number of the radiation lobe, $P_{s}$ is the signal strength of the transmitter and ω is the angle at which the radiant intensity emitted is assessed relative to the axial axis of the transmitter. The index *n* is given by the expression:

(5) $$n=-\frac{\ln\left(2\right)}{\ln\left(\cos\left(\phi_{1/2}\right)\right)}$$

where $\phi_{1/2}$ is the angle at which the signal strength is half the signal strength at $0^{\circ }$.

**Figure 2:**
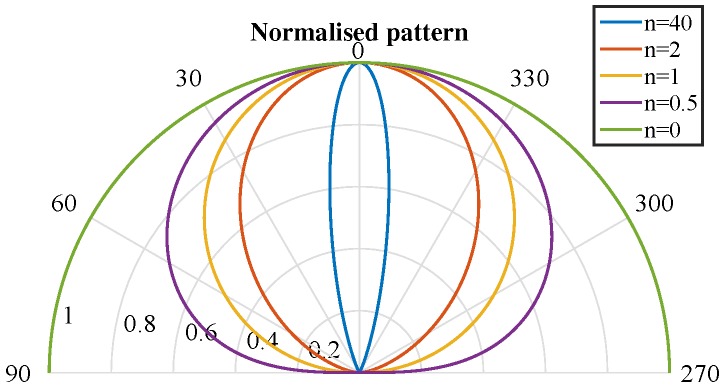
Examples of normalized radiation patterns for several values of *n*.

The index *n* characterizes transmitter directionality. Figure 2 shows diagrams of the normalized emission of a transmitter with different *n* index values. High *n* values indicate a narrow lobe, concentrating signal strength in the normal, whereas low *n* values indicate a wider lobe where signal strength is distributed in a cone with a larger aperture. When n=0, emission is isotropic, and when n=1, it is Lambertian.

The detector response relative to its axial axis, considering a thin lens, can be written as:

(6) $$R\left(\gamma \right)=\cos\left(\gamma \right)\mathrm{rect}\left(\frac{\gamma }{\mathrm{FoV}}\right)$$

If it is not possible to consider a thin lens, the cos(γ) must be replaced by a function f(γ). The rectangular function is defined as:

(7) $$\mathrm{rect}\left(x\right)=\left\{\begin{array}{cc} 1 & \mathrm{for}\left|x\right|\leq 1\\ 0 & \mathrm{for}\left|x\right|>1 \end{array}\right.$$

where FoV is the maximum angle of arrival at which the receiver is capable of receiving.

Thus, for a case that takes these last two considerations into account, without filters or thick lenses, according to Equation (3), the received signal strength can be expressed as:

(8) $$P_{\mathrm{rs}}=\frac{n_{s}+1}{2\pi }\frac{1}{d_{\mathrm{rs}}^{2}}\cos^{n_{s}}\left(\omega \right)A_{r}\cos\left(\gamma \right)P_{s}\mathrm{rect}\left(\frac{\gamma }{\mathrm{FoV}}\right)$$

If we assume that $r_{s}$ and $r_{r}$ are the coordinates where the transmitter and receiver are located, respectively, whose orientations are given by the normal vectors ${\vec{n}}_{s}$ and ${\vec{n}}_{r}$, we can calculate the angles ω and γ, as well as the distance $d_{\mathrm{rs}}$, as follows:

(9) $$\omega =\arccos\left(\frac{{\vec{n}}_{s}\cdot \left(r_{r}-r_{s}\right)}{\left|{\vec{n}}_{s}\right|\left|\left(r_{r}-r_{s}\right)\right|}\right)$$

(10) $$\gamma =\arccos\left(\frac{{\vec{n}}_{r}\cdot \left(r_{s}-r_{r}\right)}{\left|{\vec{n}}_{r}\right|\left|\left(r_{s}-r_{r}\right)\right|}\right)$$

(11) $$d_{\mathrm{rs}}=\left|r_{r}-r_{s}\right|$$

Therefore, from Equation (8) and the distance between the source and receiver $d_{\mathrm{rs}}$, the channel impulse response, considering solely the LOS component, can be expressed as:

(12) $$h\left(t\right)=P_{\mathrm{rs}}\delta \left(t-t_{\mathrm{rs}}\right)$$

where δ is the delta function and $t_{\mathrm{rs}}$ is the time it takes the signal to travel the distance between the source and the receiver, which is calculated as:

(13) $$t_{\mathrm{rs}}=\frac{d_{\mathrm{rs}}}{c}$$

where *c* is the speed of light.

### 3.3. Characterization of Reflections in the Environment

**Figure 3:**
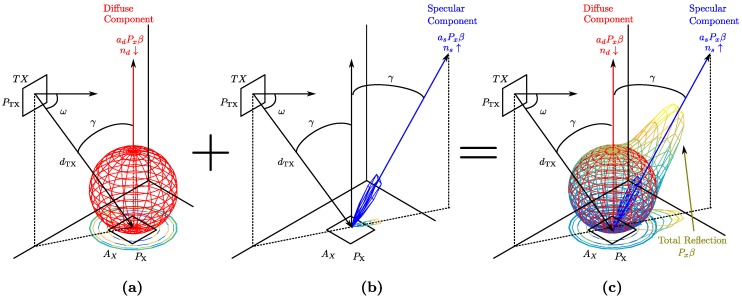
Reflection model. (**a**) Difuse component; (**b**) specular component; (**c**) total reflection.

Unlike previous studies, we will not model the signal reflections in the various elements according to a Lambertian model (proportional to $\cos\left(\theta \right)$), but according to the reflection model proposed in [4]. The latter consists of two components, one of which characterizes behaviors using a broad emission diagram, oriented according to the normal of the reflection surface, while the other characterizes behaviors using a narrower emission diagram, oriented according to the direction and course of the beam with maximum irradiance. Figure 3 gives an example of reflection at a given point *x*. The diffuse component is shown as a sphere; the specular component is shown in blue; and the total reflection is shown in shading from blue to yellow.

**Figure 4:**
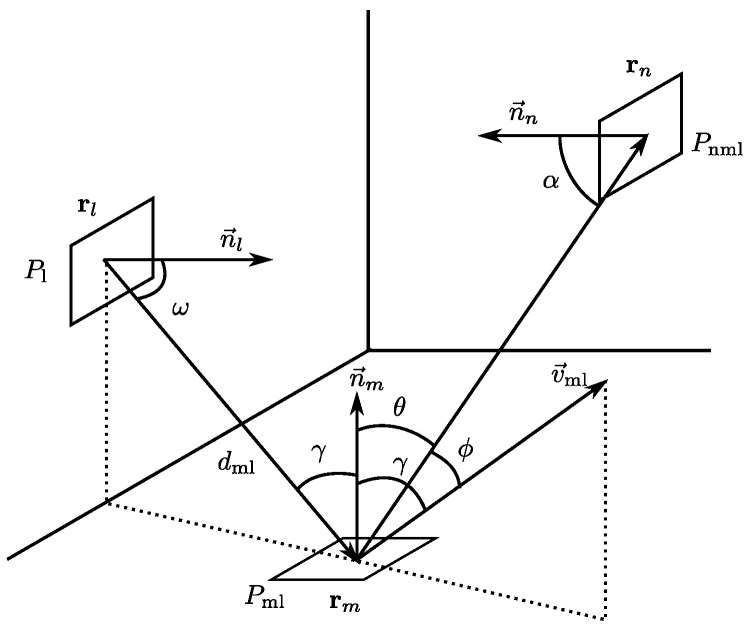
Diagram of the components of the received signal strength in element *n* due to reflection in *m* of the signal strength emitted by element *l*.

The model characterizes each surface in the environment using seven parameters, $u_{as}$, $v_{as}$, $u_{nd}$, $v_{nd}$, $u_{ns}$, $v_{ns}$ and β, which can be obtained experimentally from only 12 signal strength measurements, as shown in [4]. The proposed reflection model depends on the angle of arrival. Therefore, when calculating the received signal strength in a cell *n* from the reflection in cell *m* (following Figure 4), it is also necessary to determine the cells that previously emitted to the cell *m* in order to obtain the angles of arrival required by the model; Figure 4 gives an example of a cell receiving energy from the cell *m*, which is the cell *l*.

Consequently, the received signal strength in element *n*, due to reflection in *m* of the signal strength emitted by element *l*, is given by the expression:

(14) $$P_{\mathrm{nml}}=\left[p_{d}\left(\gamma ,\theta \right)+p_{s}\left(\gamma ,\phi \right)\right]S\cos\left(\alpha \right)\frac{1}{d_{\mathrm{nm}}^{2}}P_{\mathrm{ml}}\beta \mathrm{rect}\left(\frac{\alpha }{\mathrm{FoV}}\right)$$

where the terms $p_{d}$ and $p_{s}$ are the diffuse and specular components of the reflection model according to [4], and follow the expressions shown in Equation (15) and Equation (16), respectively:

(15) $$p_{d}\left(\gamma ,\theta \right)=\left(1-u_{\mathrm{as}}\cos^{v_{\mathrm{as}}}\left(\gamma \right)\right)\left(\frac{u_{\mathrm{nd}}\cos^{v_{\mathrm{nd}}}\left(\gamma \right)+1}{2\pi }\right)\cos^{u_{\mathrm{nd}}\cos^{v_{\mathrm{nd}}}\left(\gamma \right)}\left(\theta \right)$$

(16) $$p_{s}\left(\gamma ,\phi \right)=u_{\mathrm{as}}\cos^{v_{\mathrm{as}}}\left(\gamma \right)\left(\frac{u_{\mathrm{ns}}\cos^{v_{\mathrm{ns}}}\left(\gamma \right)+1}{2\pi }\right)\cos^{u_{\mathrm{ns}}\cos^{v_{\mathrm{ns}}}\left(\gamma \right)}\left(\phi \right)$$

α is the angle between vectors ${\vec{n}}_{n}$, and $\left(r_{m}-r_{n}\right)$ and is calculated as:

(17) $$\alpha =\arccos\left(\frac{{\vec{n}}_{n}\cdot \left(r_{m}-r_{n}\right)}{\left|{\vec{n}}_{n}\right|\left|\left(r_{m}-r_{n}\right)\right|}\right)$$

$d_{\mathrm{nm}}$ is the distance between the element *m* and the element *n*:

(18) $$d_{\mathrm{nm}}=\left|r_{n}-r_{m}\right|$$

γ is the angle between the vectors ${\vec{n}}_{m}$ and $\left(r_{l}-r_{m}\right)$ and is calculated as:

(19) $$\gamma =\arccos\left(\frac{{\vec{n}}_{m}\cdot \left(r_{l}-r_{m}\right)}{\left|{\vec{n}}_{m}\right|\left|\left(r_{l}-r_{m}\right)\right|}\right)$$

θ is the angle between the vectors ${\vec{n}}_{m}$ and $\left(r_{n}-r_{m}\right)$ and is calculated as:

(20) $$\theta =\arccos\left(\frac{{\vec{n}}_{m}\cdot \left(r_{n}-r_{m}\right)}{\left|{\vec{n}}_{m}\right|\left|\left(r_{n}-r_{m}\right)\right|}\right)$$

ϕ is the angle between the vectors $\left(r_{n}-r_{m}\right)$ and ${\vec{v}}_{\mathrm{ml}}$, where the latter is the maximum irradiance vector that can be calculated from the Householder transformation according to:

(21) $${\vec{v}}_{\mathrm{ml}}=H\left(r_{m}-r_{l}\right)$$

where H is the Householder matrix which is defined from the normal of the reflection plane (in this case, ${\vec{n}}_{m}$), as:

(22) $$H=I-\frac{2{\vec{n}}_{m}{\vec{n}}_{m}^{T}}{{\vec{n}}_{m}^{T}{\vec{n}}_{m}}$$

Therefore, the angle ϕ is obtained from the expression:

(23) $$\phi =\arccos\left(\frac{{\vec{v}}_{\mathrm{ml}}\cdot \left(r_{n}-r_{m}\right)}{\left|{\vec{v}}_{\mathrm{ml}}\right|\left|\left(r_{n}-r_{m}\right)\right|}\right)$$

*S* is the area of the cells; $P_{\mathrm{ml}}$ is the received signal strength in element *m* of the reflection in *l* from another element in the environment; and FoV is the maximum angle of arrival with respect to the normal of the element *n* that it is capable of receiving. The parameters $u_{as}$, $v_{as}$, $u_{nd}$, $v_{nd}$, $u_{ns}$, $v_{ns}$ and β are the parameters that characterize the material of element *m* defined in [4], which can be easily obtained experimentally.

## 4. Algorithm for Calculating the Impulse Response Due to Multipath

We propose using a recursive function to calculate the channel impulse response due to *K* signal rebounds in the environment, following a procedure similar to that given in [5]. The reflection in the various elements in the environment will not be considered Lambertian; instead, we will use the reflection model proposed in [4]. As a result, in order to calculate the received signal strength in a cell due to reflection, it is necessary to consider three cells: the one that emits, the one that reflects and the one that receives, not just the two cells used in [5], which complicates the method for obtaining the impulse response.

The impulse response due to multipath is given by $h\left(t\right)$ and is expressed by:

(24) $$h\left(t\right)=\sum_{k=0}^{K}h^{\left(k\right)}\left(t\right)$$

where $h^{\left(k\right)}\left(t\right)$ is the impulse response of the *k* rebound and *K* is the number of rebounds to consider. In other words, k=0 is the LOS path, k=1 is the impulse response due to one rebound, k=2 is the impulse response due to two rebounds, and so on successively until the reflection *K*. The higher the *K*, the closer to reality, but the computational load for calculation will also rise.

Two functions must be used to obtain each $h^{\left(k\right)}\left(t\right)$. First, to calculate the response to the impulse from the source element in each of the cells in the environment, the following function is used:

(25) $$h_{0}\left(t;s,r\right)=P_{rs}\delta \left(t-t_{\mathrm{rs}}\right)$$

where *s* and *r* are the source and receiver elements, $P_{rs}$ is the signal strength received by element *r* from *s*, which is calculated according to the expression (8), and $t_{\mathrm{rs}}$ is the signal delay between the two elements, calculated according to Equation (13).

The second function is used to calculate the impulse response to the signal emitted from an element *l*, rebounded off an element *m* and received by an element *n* and has the form:

(26) $$h_{r}\left(t;l,m,n\right)=P_{nml}\delta \left(t-t_{\mathrm{nm}}\right)$$

where $P_{nml}$ is calculated according to Equation (14) and the delay $t_{\mathrm{nm}}$ is obtained from the distance between the elements *m* and *n* and the speed of light.

The impulse response due to the first rebound $h^{\left(1\right)}\left(t\right)$, where *s* is the source element and *r* is the receiving element, is calculated as:

(27) $$h^{\left(1\right)}\left(t\right)=\sum_{m=1}^{N}\left[h_{0}\left(t;s,m\right)*h_{r}\left(t-t_{ms};s,m,r\right)\right]$$

where the response is obtained to the impulse from the source element to each *m* element and is convolved with the response to the impulse obtained when it is emitted by the source, rebounded off element *m* and received at the receiver, delayed by time $t_{ms}$ due to the distance between the source and the element *m*. *N* is the total number of elements forming the environment.

Analogously, one can obtain the impulse response due to the second rebound $h^{\left(2\right)}\left(t\right)$. In this case, after the signal strength from the source is received in element *m*, it is emitted again from this element to all *n* elements in the environment, and finally from these to the receiver. This can be calculated as:

(28) $$h^{\left(2\right)}\left(t\right)=\sum_{m=1}^{N}\left[h_{0}\left(t;s,m\right)*\sum_{n=1}^{N}\left[h_{r}\left(t-t_{ms};s,m,n\right)*h_{r}\left(t-t_{nm}-t_{ms};m,n,r\right)\right]\right]$$

To obtain the impulse response due to the third rebound $h^{\left(3\right)}\left(t\right)$, it is assumed that after passing through all of the *n* elements, the signal is emitted to all *l* elements, taking into account that it comes from *m* elements and finally reaches the receiver. This is calculated as follows:

(29) $$h^{\left(3\right)}\left(t\right)=\sum_{m=1}^{N}\left[h_{0}\left(t;s,m\right)*\sum_{n=1}^{N}\left[h_{r}\left(t-t_{ms};s,m,n\right)*\sum_{l=1}^{N}\left[h_{r}\left(t-t_{nm}-t_{ms};m,n,l\right)*h_{r}\left(t-t_{ln}-t_{nm}-t_{ms};n,l,r\right)\right]\right]\right]$$

To obtain the impulse response for the *k* rebound, a similar procedure is employed considering *k* rebounds.

In the expressions given above, time is considered to be continuous. However, as discussed earlier, in order to implement the algorithm, it is necessary to discretize time.

Therefore, we generated a recursive function to obtain the impulse response for rebound *k* of the signal in the environment. The total impulse response will be the sum of the impulse responses from the rebound k=0 (LOS paths) to the rebound k=K, according to the Equation (24).

In Algorithm 1, we give the pseudocode for this recursive function.

**Algorithm 1** Recursive algorithm to calculate the impulse response.
1:**function** h_func($h,\;\mathrm{tx},\;\mathrm{rx},\;s,\;s_{\mathrm{ant}},\;P,\;d,\;k_{i},\;k$)2:      **if** $k==k_{i}$ **then**                                              ▹ Source is TX3:             **if** $k_{i}==0$ **then**                                        ▹ Receiver is RX4:                    Calculate [ω, γ, $d_{\mathrm{rs}}]$ from (**tx, rx**) with (9)–(11)5:                    Calculate signal strength $P_{\mathrm{rs}}$ with (8) substituting $P_{s}$ for P6:                    Calculate discrete time $t_{\mathrm{rs}}$ from distance $d+d_{\mathrm{rs}}$, and light velocity7:                    **if** γ < FoV **then**8:                          $h\left(t_{\mathrm{rs}}\right)=h\left(t_{\mathrm{rs}}\right)+P_{\mathrm{rs}}$9:                    **end if**10:             **else**                              ▹ Received signal strength at all points from TX11:                    **for** i from 1 to N **do**12:                         Calculate [ω, γ, $d_{\mathrm{rs}}]$ from $\left(\mathrm{tx},i\right)$ with (9)–(11) substituting rx for the i element13:                         Calculate signal strength $P_{\mathrm{rs}}$ with (8) substituting rx for the i element and Ps for P14:                         h_func($h,\;\mathrm{tx},\;\mathrm{rx},\;i,\;\mathrm{tx},\;P_{\mathrm{rs}},\;d+d_{\mathrm{rs}},\;k_{i}-1,\;k$)15:                    **end for**16:             **end if**17:      **else**                                              ▹ Source is not TX18:             **if** $k_{i}==0$ **then**                                     ▹ Receiver is RX19:                    Calculate [α, $d_{\mathrm{nm}},$ γ,θ,ϕ] from $\left(s_{\mathrm{ant}},s,\mathrm{rx}\right)$ with (17)–(20), (23) substituting n=rx, m=s and $l=s_{\mathrm{ant}}$20:                    Calculate signal strength $P_{\mathrm{nml}}$ with (14) substituting $P_{\mathrm{ml}}$ for P21:                    Calculate discrete time $t_{\mathrm{nm}}$ from distance $d+d_{\mathrm{nm}}$, and light velocity22:                    **if** α < FoV **then**23:                         $h\left(t_{\mathrm{nm}}\right)=h\left(t_{\mathrm{nm}}\right)+P_{\mathrm{nml}}$24:                    **end if**25:             **else**                             ▹ Received signal strength at all points from TX26:                    **for** i from 1 to N **do**27:                         Calculate [α,$d_{\mathrm{nm}},$γ,θ,ϕ] from $\left(s_{\mathrm{ant}},s,i\right)$ with (17)–(20), (23) substituting n=i, m=s and $l=s_{\mathrm{ant}}$28:                         Calculate signal strength $P_{\mathrm{nml}}$ with (14) substituting $P_{\mathrm{ml}}$ for P29:                         h_func($h,\;\mathrm{tx},\;\mathrm{rx},\;i,\;s,\;P_{\mathrm{nml}},\;d+d_{\mathrm{nm}},\;k_{i}-1,\;k$)30:                    **end for**31:             **end if**32:      **end if**33:**end function**


*h* is the impulse response; tx is the transmitter; rx is the receiver; *s* is the element that reflects the light from $s_{\mathrm{ant}}$; *P* is the signal strength; *d* is the distance; *k* is the number of rebounds from which to obtain the impulse response; and $k_{i}$ is a parameter that indicates the rebound for each iteration of the recursive algorithm. The function (in the algorithm) is called the value of $k_{i}=k$. Each element *i* in the array *h* corresponds to the signal strength received by the detector for each instant of time $t=i*\Delta_{t}$.

**Figure 5:**
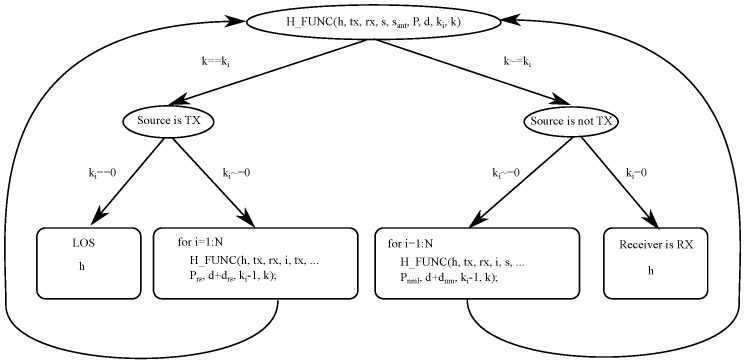
Diagram of the recursive algorithm used to obtain the impulse response.

A diagram of the recursive algorithm is shown in Figure 5.   

For AoA-based systems, in which time and hence signal phase information is not relevant, a modification of the proposed algorithm can be used. In this case, the algorithm calculates the signal strength that would reach the receiver from each of the elements in the environment after *K* rebounds. The implementation is as follows. The array *h* has a size equal to the number of elements in the environment. Therefore, the value of element *i* of *h* will correspond to the signal strength received by the detector of element *i* in the environment. The algorithm is run as in the previous case, but without taking time into account.

The algorithm for AoA systems is given in Algorithm 2.

**Algorithm 2** Recursive algorithm to calculate the received signal strength at the receiver from each element in the environment.
1:**function** h_func_aoa($h,\;\mathrm{tx},\;\mathrm{rx},\;s,\;s_{\mathrm{ant}},\;P,\;d,\;k_{i},\;k$)2:      **if** $k==k_{i}$ **then**                                             ▹ Source is TX3:             **if** $k_{i}==0$ **then**                                       ▹ Receiver is RX4:                    Calculate [ω, γ, $d_{\mathrm{rs}}]$ from (**tx, rx**) with (9)–(11)5:                    Calculate signal strength $P_{\mathrm{rs}}$ with (8) substituting $P_{s}$ for P6:                    **if** γ < FoV **then**7:                          $h_{\mathrm{LOS}}=P_{\mathrm{rs}}$8:                    **end if**9:             **else**                            ▹ Received signal strength at all points from TX10:                    **for** i from 1 to N **do**11:                         Calculate [ω, γ, $d_{\mathrm{rs}}]$ from $\left(\mathrm{tx},i\right)$ with (9)–(11) substituting rx for the i element12:                         Calculate signal strength $P_{\mathrm{rs}}$ with (8) substituting rx for the i element and Ps for P13:                         h_func_aoa($h,\;\mathrm{tx},\;\mathrm{rx},\;i,\;\mathrm{tx},\;P_{\mathrm{rs}},\;d+d_{\mathrm{rs}},\;k_{i}-1,\;k$)14:                    **end for**15:             **end if**16:      **else**                                              ▹ Source is not TX17:             **if** $k_{i}==0$ **then**                                     ▹ Receiver is RX18:                    Calculate [α,$d_{\mathrm{nm}},$γ, θ, ϕ] from $\left(s_{\mathrm{ant}},s,\mathrm{rx}\right)$ with (17)–(20), (23) substituting n=rx, m=s and $l=s_{\mathrm{ant}}$19:                    Calculate signal strength $P_{\mathrm{nml}}$ with (14) substituting $P_{\mathrm{ml}}$ for P20:                    **if** α < FoV **then**21:                    $h\left(s_{i}\right)=h\left(s_{i}\right)+P_{\mathrm{nml}}$22:                    **end if**23:             **else**                         ▹ Received signal strength at all points from TX24:                    **for** i from 1 to N **do**25:                    Calculate [α,$d_{\mathrm{nm}},$γ,θ,ϕ] from $\left(s_{\mathrm{ant}},s,i\right)$ with (17)–(20), (23) substituting n=i, m=s and $l=s_{\mathrm{ant}}$26:                    Calculate signal strength $P_{\mathrm{nml}}$ with (14) substituting $P_{\mathrm{ml}}$ for P27:                    h_func_aoa($h,\;\mathrm{tx},\;\mathrm{rx},\;i,\;s,\;P_{\mathrm{nml}},\;d+d_{\mathrm{nm}},\;k_{i}-1,\;k$)28:                    **end for**29:             **end if**30:      **end if**31:**end function**


As can be seen, the algorithm is the same except that the meaning of *h* has changed and $s_{i}$ has been added, which corresponds to the index of the element *s*.

As this is a recursive function, the run time depends on the number of elements into which the environment has been divided and the number of successive reflections considered; it is proportional to $N^{k}$, where *N* is the total number of elements and *k* is the rebound considered. The total time would be:

(30) $$t_{\mathrm{RUN}}\propto \sum_{k=0}^{K}N^{k}$$

Table 1 gives the number of indirect paths considered for a 3 × 3 × 3 m environment, in which there may be reflections from six sides, depending on the size *S* of the cell and the number of *k* rebounds considered.

Programmed in C in a single threading on a PC with an Intel Core 2 Duo E8400 and 8.00 GB DDR2 RAM, the algorithm requires about $3.3207\times 10^{-6}$ s to run each multipath. Table 2 gives approximate run times depending on *k* and *S*.

This algorithm can be used to simulate different types of signal by changing the equations for the signal strength emitted, reflected and received that characterize the type of signal to analyze.

## 5. Validation of the Procedure

In this section, we will analyze the results of the proposed algorithm and procedure for estimating and calculating the indirect paths, indicating the effects on different applications, using an IR signal. To validate our model, we conducted an empirical test in a controlled environment and observed the signal received at the receiver due to multipath and the effects of these on measurements. Then, using the model described here, we simulated the same environment conditions that had been tested empirically and compared the results. We also compared them with those obtained using the traditional model proposed in the references cited.

### 5.1. Characteristics of the Tests

Table 3 gives the most important characteristics of the environment, the transmitter and the receiver used in the tests.

The transmitter and receiver were placed in a space consisting of two walls perpendicular to each other (with an “L” shape, made up by orthogonal planes with equations z = 1 and x = 0), in which the light beams are reflected and reach the receiver (with the first and second rebound components); the other two walls did not exist, assuming that they did not produce rebounds. The detector was positioned in such a way that it could be assumed that there was no wall where it was placed (there were no rebounds in this part of the environment either). The detector was static, but the transmitter was mobile and occupied different locations so that we could assess the behavior in various positions. The dimensions of the environment and the orientation between the transmitter and receiver are shown in Table 3, and were selected so as to ensure, given the FoV of the detector, that the signal due to the direct path was null.

**Figure 6:**
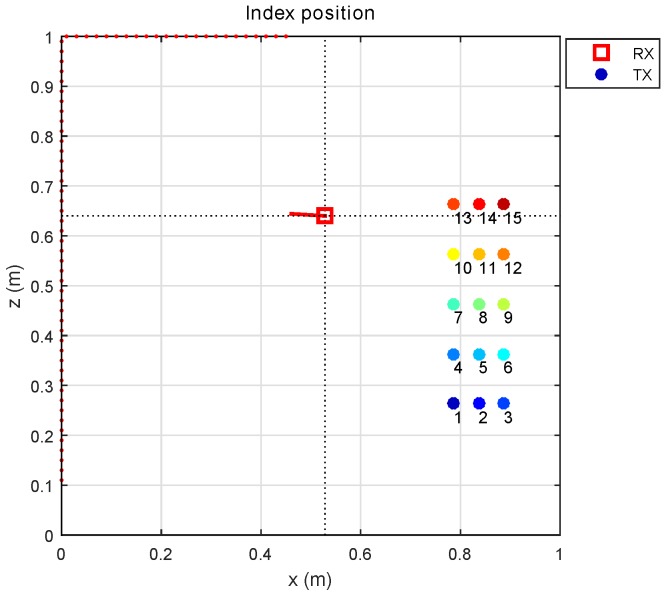
Transmitter positions within the environment.

The transmitter was placed in a total of 15 positions, as shown in Figure 6. The indices of the transmitter positions are indicated by differently colored circles and differentley numbers.

**Figure 7:**
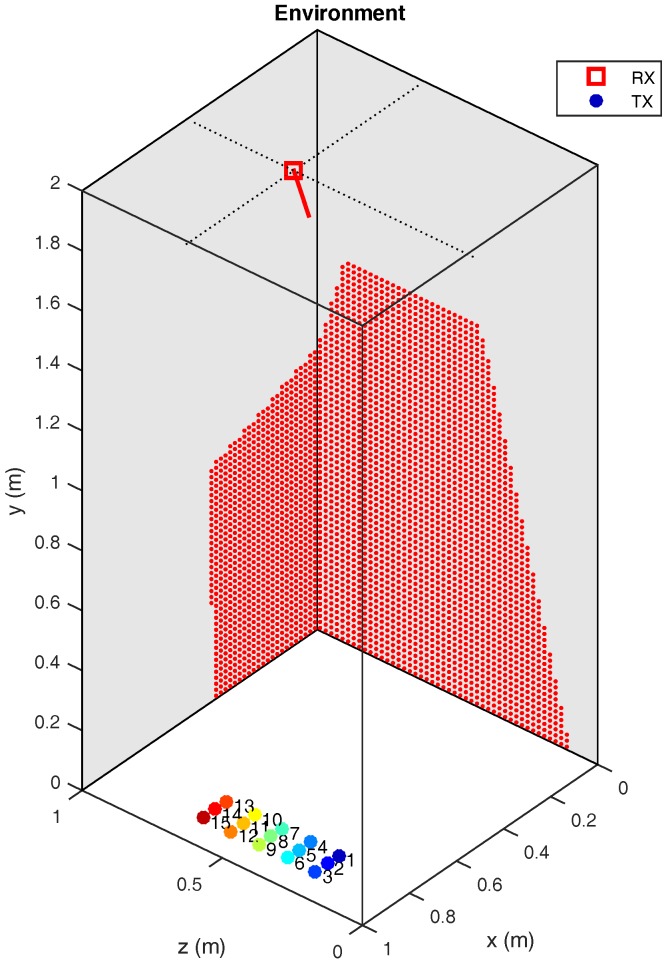
Diagram of the environment and the position of the transmitter and receiver.

Figure 7 shows a diagram of the test environment. The transmitter positions are indicated by differently colored circles, and the detector position is indicated by a red square. The red dots indicate the elements in the environment within the FoV of the detector. Only reflections on the walls marked in grey were considered (we did not consider reflections on the plane y = 2 m, which is also grey due to perspective in the figure).

**Figure 8:**
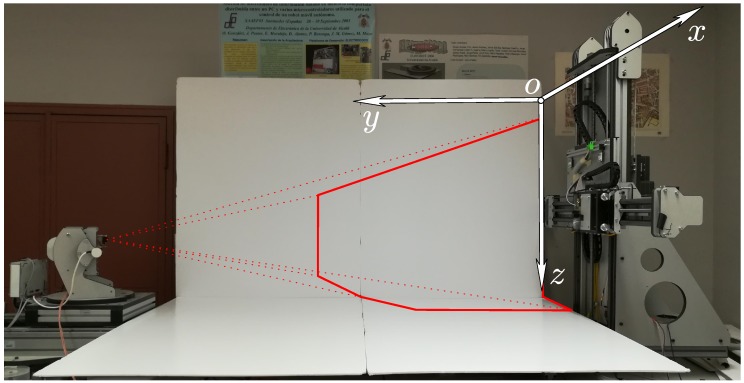
Photograph of the bench on which the environment was mounted.

Figure 8 shows a photograph of the bench on which the environment was mounted in order to control the angles and movements of the transmitter. Also, FoV of the detector is shown in red.

**Figure 9:**
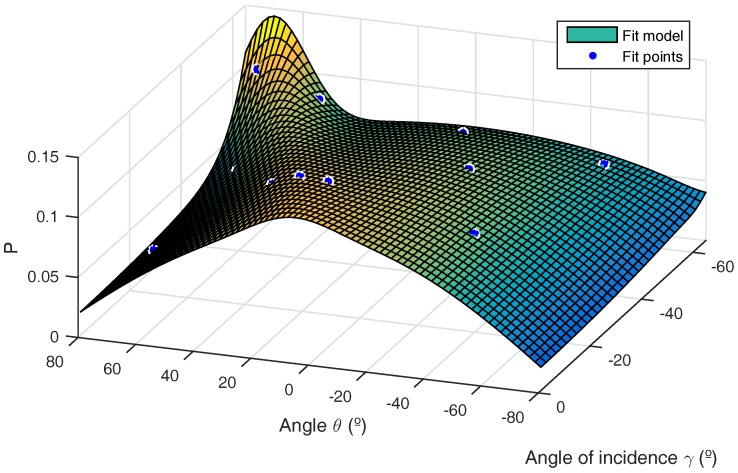
Foam board reflection model.

The materials used to construct the environment consisted of 5 mm-thick foam boards. To determine the characteristics of reflection in foam boards, we followed the procedure indicated in [4]. Thus, from 12 simple measurements, we obtained the reflection model parameters to enter into our multipath behavior model. Figure 9 indicates the behavior of reflections in this material. The surface shows the reflection model, fitting the parameters for the 12 points given in blue. Table 4 gives the values obtained for the parameters. Note that this material does not present a significant “s” component according to the model in [4].

The scenario used was selected for ease of implementation and explanation of the process, but could be applied to spaces of any shape, provided that there are no shaded areas within the environment (the possibility of working with shaded areas and objects in the environment will be addressed shortly).

The simulations took into account two rebounds using a $5\times 5\;{\mathrm{cm}}^{2}$ grid.

The receiver was placed in the position and orientation indicated in Table 3, controlled by the test bench positioner, which presented errors of 1 mm and $0.1^{\circ }$ for the displacement and rotation step, respectively. We used a position-sensitive device (PSD) as the receiver [17]. The four output currents of this type of photodetector are used to obtain the point of impact of the optical signal received on the sensor surface. In an optical system, this information can be used to calculate the angle of arrival of the received signal. The receiver was a PSD S5991-01 with a 1-inch, 15.5-mm focal length lens. For the simulation, we considered a PSD with an area of 9 × 9 mm and a similar lens.

### 5.2. Results of Empirical Tests

Given the implementation conditions of the test, the detector should not receive any information, due to the absence of a direct signal path. Therefore, the received signal and its measurements will be errors resulting from multipath.

The procedure followed in the tests was as follows. The transmitter was placed in each of the 15 positions indicated in Figure 6 and emitted a sinusoidal optical signal of 50 kHz. The signal received by the PSD was acquired at a sampling rate of 10 Msamples/s. This signal was composed solely of the different signal reflections in the environment. Once the signal had been acquired, a bandpass filter was applied, centered at 50 kHz, to eliminate as much noise as possible. From these values and after applying electrical [18] and geometric [19] corrections, we obtained the points of impact on the surface of the PSD.

**Figure 10:**
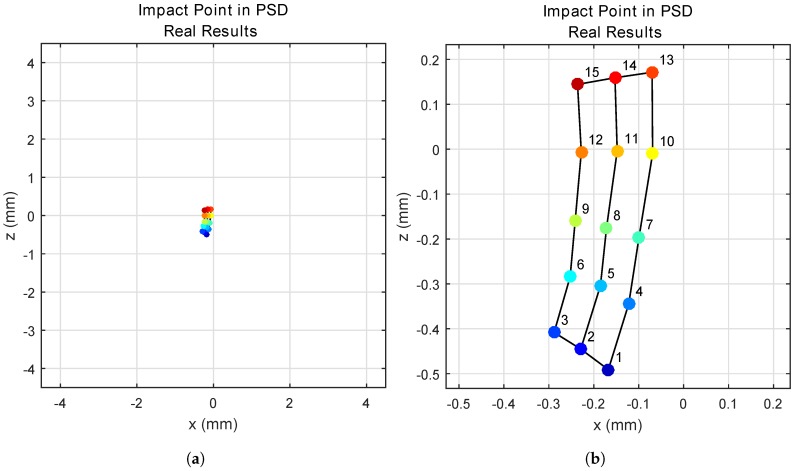
Points of impact on the PSD surface, determined from real measurements. (**a**) Total position-sensitive device (PSD) size; (**b**) enlarged image.

Figure 10 shows the points of impact on the surface of the PSD for each transmitter position. Figure 10a shows the points of impact on the 9 × 9 mm surface of the PSD, and Figure 10b gives an enlarged image of the area.

Although the errors in position determination may seem small in the image of the sensor surface, for the case of Positions 3 and 15, for example, the errors in determining the source at 2 m would be (51.6 mm, 38.5 mm) and (19.3 mm, 31 mm), respectively. The greater the distance, the greater the errors. In addition, if it were necessary take the phase into account (as in differential phase of arrival (DPoA)), the error would increase to several tens of centimeters.

### 5.3. Results Obtained with the Proposed Model and Comparison with Empirical Tests

Following the empirical tests, we simulated the same environment using the proposed model and the information given in Section 5.1.

We calculated the signal strength that reached all of the cells into which the environment was divided within the FoV of the PSD. In this case, we did not consider the signal phase since only the information on signal strength was required. We calculated the signal strength that reached each cell after *K* rebounds, and then, considering each cell as a transmitter, we obtained the strength and point of impact of the signal reaching the PSD via the lens system. We assumed that there were no distortions in the lens or the PSD and that the only errors were those due to multipath. This was a reasonable assumption given that such errors had been corrected in the experimental tests by electrical and geometric calibration. Note that in this case, the errors were given by the images formed in the PSD of the beams that arrived by non-LOS paths (multipath).

Once the signal strength and point of impact of each of the multipaths has been determined, the center of mass of all of the points can be obtained. The center of mass is the point at which the PSD returns.

**Figure 11:**
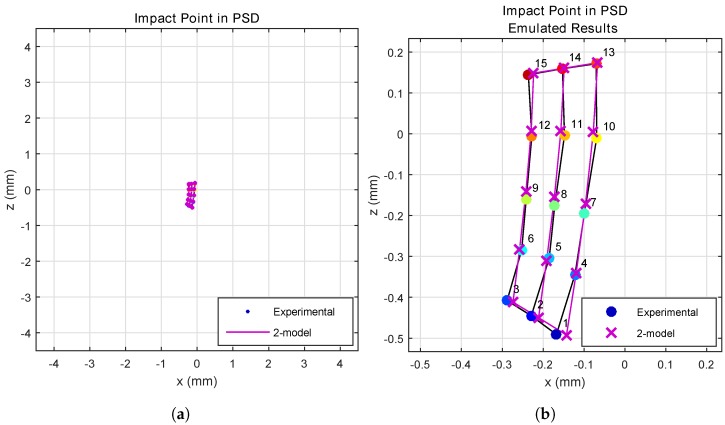
Points of impact on the PSD surface, determined from real and simulated measurements. (**a**) Total PSD size; (**b**) enlarged image (**a**).

In Figure 11, the results obtained with a 5-cm cell are compared with the empirical results. Figure 11a shows this for total PSD size, while Figure 11b shows an enlarged image.

As can be seen, the results obtained from empirical tests and our model were very similar, with only minor differences. These latter may have been due to small errors in positioning or in position determination on the test bench.

**Figure 12:**
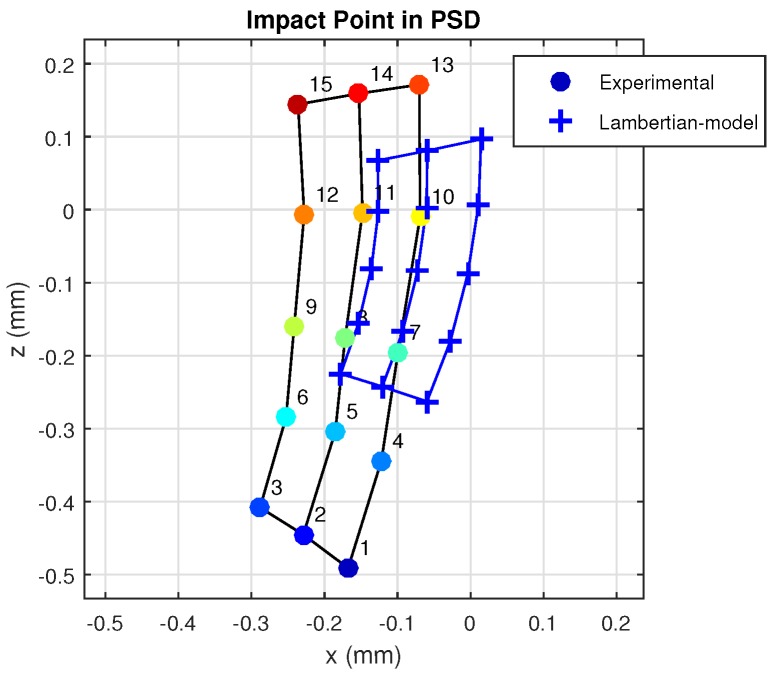
Points of impact on the PSD surface, obtained from real and simulated measurements using a Lambertian reflection model.

In Figure 12, we will compare the empirical results with the results that would have been obtained using the model proposed in previous studies by other researchers (Lambertian).

As can be seen, the differences between the results obtained in reality and using an earlier model to the one proposed here are quite significant. Note also that this even occurs with a material with a small “s” component [4]. If it were a material with a more specular behavior, these differences would be very large.

**Figure 13:**
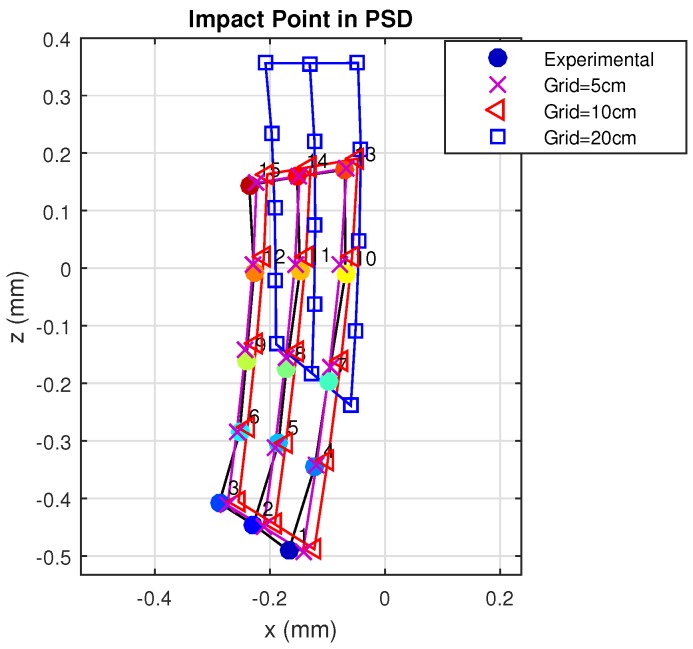
Points of impact on the PSD surface according to grid size, determined from real and simulated measurements.

The previous simulations were performed considering two rebounds and a cell size of 5 cm. Below, we describe simulations performed using three different cell size values to discretize the environment. Evidently, the smaller the cell size, the closer the behavior will be to the real case. Figure 13 gives the results for the three different cell sizes.

As can be seen, when the cell size was less than 10 cm, the model and procedure yielded results that were very similar and close to the real case. However, when cell size was increased to 20 cm, the behavior of the discretized model was no longer close to reality.

Setting up a threshold may depend on many factors, such as the geometry and materials of the environment and characteristics of both the emitter and receiver. In any case, as the surface of the grid increases, the results will become less realistic because the irradiance reaching all points on the surface of each cell is no longer similar, and the hypothesis on which is based our approach, considering that the continuous space behavior is correctly modeled by a discrete grid space, is no longer fulfilled.

In general, it can be considered that the irradiance reaching the entire surface of each grid cell is constant when the side of the grid cell is at least 10-times lower than the distance that separates it from the light source.

In any case, we set up the threshold for the size grid cells as 20-times smaller than the minimum distance between emitter and receiver (i.e., 10 cm × 10 cm for 2-m distance), which provides almost identical results between the empirical tests and the proposed model, with an affordable computational load.

With this cell size, we choose the best balanced solution. In addition, we should comment that reducing this size value does not significantly improve the results, but does greatly increase the computation time.

Table 5 shows the run time for these tests using a PC with an Intel Core 2 Duo E8400 and 8.00 GB DDR2 RAM.

It should be noted that performing very precise behavioral tests in controlled environments is simple if the environments have a regular structure (geometrically simple) and can be emulated on a workbench, but extraordinarily complicated in more complex environments. The empirical tests carried out on a workbench have yielded results similar to the one shown here.

Performing real tests in controlled complex environments is very difficult and expensive to carry out. Nevertheless, current works make use of this modeling tool to model the MP errors for indoor positioning of mobile agents. Such positioning error tests in complex environments are much easier to obtain empirically than the behavior of the proposed model. In fact, it has been proven that based on the model proposed here, the positioning errors can be emulated, but its extension will be shown in a future work in which the geometry is considered as known (e.g., indoor environments for mobile agents), as well as the position of detector, in order to evolve towards a statistical model in a future work.

## 6. Conclusions and Future Works

We conclude that the continuous behavior of the reflections and multipath can be simulated using a discretized procedure, if certain conditions and constraints are met. We found that the proposed model and procedure presented a perfect fit to real behavior and made it possible to accurately obtain the impulse response; however, it is still necessary to use a model of the light reflected by more complex materials than those that have traditionally been used.

Initially, the method had been designed for its use in rigid structured environments (floor, walls and ceiling form a cube space) considering also that there is an LOS path and other reflections between the emitter and receiver. This proposal assumes that the reflection characteristics of the materials must be known a priori or obtained using [4]. A dynamic environment would constantly modify reflections and therefore the MP. Something similar would happen if there exist shadow areas for LOS and reflection paths.

Theoretically, we could analyze these kinds of environments considering shadow zones among different cells. It would be a more laborious work with a quite high computational cost. However, with the current version, it is possible to characterize any environment in an approximated way, obtaining very realistic results of MP effects without requiring their physical implementation.

Having verified that the model is capable of reproducing the behavior of the environment, we conclude that it can be used in many applications (in communications and local positioning systems, for example) to estimate the problems of these types of system due to multipath without the need to implement them to determine their behavior.

In communications, the impulse response can be used to quantify the time dispersion of multipath channels.

For local positioning systems, it is necessary to distinguish between the type of system. PoA- or DPoA-based LPS require impulse response information, while in AoA-based systems, it is necessary to know the signal strength reaching the detector from each element in the environment after *K* signal rebounds in the same.

In AoA-based systems using a PSD, for example, it is possible to obtain the error introduced by multipath by comparing the angle of arrival due solely to LOS paths with that obtained after considering *K* signal rebounds.

This model and tool will be used in future works to model errors due to multipath in indoor positioning (using an infrared signal) in order to reduce its influence. In the first step, the space geometry and the position of the detector will be considered as known; this is the case of mobile robots moving inside indoor environments.

## Figures and Tables

**Table 1:** Number of indirect paths depending on cell size and the number of rebounds.

*S*	*N*	k=1	k=2	k=3
$1\times 1\;m^{2}$	54	54	2916	157,464
$0.5\times 0.5\;m^{2}$	216	216	46,656	10,077,696
$0.25\times 0.25\;m^{2}$	864	864	746,496	644,972,544
$0.1\times 0.1\;m^{2}$	5400	5400	29,160,000	$1.5746\times 10^{+11}$

**Table 2:** Run times depending on cell size and the number of rebounds.

*S*	*N*	k=1	k=2	k=3
$1\times 1\;m^{2}$	54	0.179 ms	9.683 ms	0.523 s
$0.5\times 0.5\;m^{2}$	216	0.717 ms	0.1549 s	33.46 s
$0.25\times 0.25\;m^{2}$	864	2.87 ms	2.479 s	35 min 41.7 s
$0.1\times 0.1\;m^{2}$	5400	17.9 ms	1 min 36.8 s	6 days 1 h 14 min 37 s

**Table 3:** Characteristics of the environment, the transmitter and the receiver.

Room and Surface	Source	Receiver
Length (x): 1 m	x: Figure 6	x: 0.5 m
Height (y): 2 m	y: 0 m	y: 2 m
Width (z): 1 m	z: Figure 6	z: 0.64 m
Reflecting coefficients:	Lambertian mode: 1	Area: $5.0671\times 10^{-4}$ $m^{2}$
Parameters: Table 4	Area vector: [0 1 0]	Area vector: [−0.339 0.940 0.021]
FoV: $90^{\circ }$	Transmitted Power: 1 W	FoV: $\pm 16^{\circ }$

**Table 4:** Foam board reflection model parameters.

$u_{\mathrm{as}}$	$v_{\mathrm{as}}$	$u_{\mathrm{nd}}$	$v_{\mathrm{nd}}$	$u_{\mathrm{ns}}$	$v_{\mathrm{ns}}$	β
0.01175	−2.194	0.9738	1.002	19.14	−0.163	0.3447

**Table 5:** Run times depending on grid size.

Grid	$5\times 5\;{\mathrm{cm}}^{2}$	$10\times 10\;{\mathrm{cm}}^{2}$	$20\times 20\;{\mathrm{cm}}^{2}$
**Run Times**	8.49 s	0.53 s	0.033 s
